# Ascle—A Python Natural Language Processing Toolkit for Medical Text Generation: Development and Evaluation Study

**DOI:** 10.2196/60601

**Published:** 2024-10-03

**Authors:** Rui Yang, Qingcheng Zeng, Keen You, Yujie Qiao, Lucas Huang, Chia-Chun Hsieh, Benjamin Rosand, Jeremy Goldwasser, Amisha Dave, Tiarnan Keenan, Yuhe Ke, Chuan Hong, Nan Liu, Emily Chew, Dragomir Radev, Zhiyong Lu, Hua Xu, Qingyu Chen, Irene Li

**Affiliations:** 1 Centre for Quantitative Medicine Duke-NUS Medical School Singapore Singapore; 2 Department of Linguistics Northwestern University Evanston, IL United States; 3 Department of Computer Science Yale University New Haven, CT United States; 4 Yale School of Public Health Yale University New Haven, CT United States; 5 Yale New Haven Hospital Yale School of Medicine Yale University New Haven, CT United States; 6 Division of Epidemiology and Clinical Applications National Eye Institute, National Institutes of Health Bethesda, MD United States; 7 Department of Anesthesiology Singapore General Hospital Singapore Singapore; 8 Department of Biostatistics and Bioinformatics Duke University Durham, NC United States; 9 Program in Health Services and Systems Research Duke-NUS Medical School Singapore Singapore; 10 Institute of Data Science National University of Singapore Singapore Singapore; 11 National Center for Biotechnology Information, National Library of Medicine National Institutes of Health Bethesda, MD United States; 12 Department of Biomedical Informatics and Data Science Yale School of Medicine Yale University New Haven, CT United States; 13 Information Technology Center University of Tokyo Kashiwa Japan; 14 Smartor LLC Tokyo Japan

**Keywords:** natural language processing, machine learning, deep learning, generative artificial intelligence, large language models, retrieval-augmented generation, healthcare

## Abstract

**Background:**

Medical texts present significant domain-specific challenges, and manually curating these texts is a time-consuming and labor-intensive process. To address this, natural language processing (NLP) algorithms have been developed to automate text processing. In the biomedical field, various toolkits for text processing exist, which have greatly improved the efficiency of handling unstructured text. However, these existing toolkits tend to emphasize different perspectives, and none of them offer generation capabilities, leaving a significant gap in the current offerings.

**Objective:**

This study aims to describe the development and preliminary evaluation of Ascle. Ascle is tailored for biomedical researchers and clinical staff with an easy-to-use, all-in-one solution that requires minimal programming expertise. For the first time, Ascle provides 4 advanced and challenging generative functions: question-answering, text summarization, text simplification, and machine translation. In addition, Ascle integrates 12 essential NLP functions, along with query and search capabilities for clinical databases.

**Methods:**

We fine-tuned 32 domain-specific language models and evaluated them thoroughly on 27 established benchmarks. In addition, for the question-answering task, we developed a retrieval-augmented generation (RAG) framework for large language models that incorporated a medical knowledge graph with ranking techniques to enhance the reliability of generated answers. Additionally, we conducted a physician validation to assess the quality of generated content beyond automated metrics.

**Results:**

The fine-tuned models and RAG framework consistently enhanced text generation tasks. For example, the fine-tuned models improved the machine translation task by 20.27 in terms of BLEU score. In the question-answering task, the RAG framework raised the ROUGE-L score by 18% over the vanilla models. Physician validation of generated answers showed high scores for readability (4.95/5) and relevancy (4.43/5), with a lower score for accuracy (3.90/5) and completeness (3.31/5).

**Conclusions:**

This study introduces the development and evaluation of Ascle, a user-friendly NLP toolkit designed for medical text generation. All code is publicly available through the Ascle GitHub repository. All fine-tuned language models can be accessed through Hugging Face.

## Introduction

Medical texts pose considerable challenges due to their domain-specific nature, including issues such as ambiguities, frequent abbreviations, and specialized terminology [1,2]. The manual curation of these texts is both time-consuming and labor-intensive [2]. Therefore, natural language processing (NLP) algorithms have been developed to automate text processing [2-4]. Recent years have seen a notable shift toward the use of domain-specific pretrained language models, transitioning from shallow embeddings such as BioWordVec [5] and BioSentVec [6] to advanced architectures like Bidirectional Encoder Representations from Transformers (BERT) [7], such as BioBERT [8], ClinicalBERT [9], and PubMedBERT [10]. Furthermore, large language models (LLMs), such as Med-PaLM [11] and Med-Gemini [12], have demonstrated powerful generative capabilities, possessing exceptional zero- and few-shot performance. These domain-specific language models have substantially enhanced the effectiveness of NLP tasks in the biomedical and clinical domains [13-15].

Despite the success of these advanced methods, their complexity remains a significant barrier to practical application for health care professionals lacking basic programming skills. Consequently, there is an increasing demand for user-friendly and accessible toolkits designed to simplify medical text processing. Multiple toolkits for text processing are available in the biomedical domain. Table 1 summarizes representative tools. While there are many other useful tools, here we mainly limit our comparison with Python-based open-source toolkits.

**Table 1:** A comparison of Ascle with existing Python-based toolkits.

Toolkits	Question- Answering^a^	Text Summarization	Text Simplification	Machine Translation	Basic NLP^b^ Functions	Query Search
MIMIC-Extract [16]	—^c^	—	—	—	—	✓
ScispaCy [17]	—	—	—	—	✓	—
MedspaCy [18]	—	—	—	—	✓	—
Transformers-sklearn [19]	—	—	—	—	✓	—
Stanza Biomed [20]	—	—	—	—	✓	—
Ascle (this study)	✓	✓	✓	✓	✓	✓

^a^For the question-answering task, we specifically propose a retrieval-augmented generation framework for large language models that incorporates a medical knowledge graph with ranking techniques.

^b^NLP: natural language processing. Basic natural language processing functions include abbreviation extraction, sentence tokenization, word tokenization, negation detection, hyponym detection, Unified Medical Language System concept extraction, named entity recognition, document clustering, part-of-speech tagging, entity linking, text summarization (extractive methods), and multiple-choice question-answering. It is worth noting that not every toolkit includes these 12 basic natural language processing functions, but Ascle includes them all.

^c^Not applicable.

These existing toolkits tend to emphasize different perspectives, and none of them offer generation capabilities, leaving a significant gap in the current offerings. In response, we present Ascle, a pioneering NLP toolkit for medical text generation, which, for the first time, includes 4 advanced generative functions. We fine-tuned 32 domain-specific language models and evaluated them thoroughly on 27 established benchmarks. In addition, for the question-answering task, we developed a retrieval-augmented generation (RAG) framework [21] that combines a medical knowledge graph (The Unified Medical Language System [UMLS]) [22] with ranking techniques, aimed at improving the reliability of long-form answers [15]. We uploaded all fine-tuned language models to Hugging Face and listed 32 fine-tuned language models along with 27 benchmarks in Multimedia Appendix 1 for a clearer explanation.

In conclusion, Ascle empowers a diverse spectrum of users, from novices to experienced professionals, enabling them to effortlessly address their NLP tasks, even with limited technical expertise in handling textual data. We believe that Ascle not only democratizes access to cutting-edge methods but also expedites their integration into health care.

## Methods

### The Overall Architecture of Ascle

**Figure 1:**
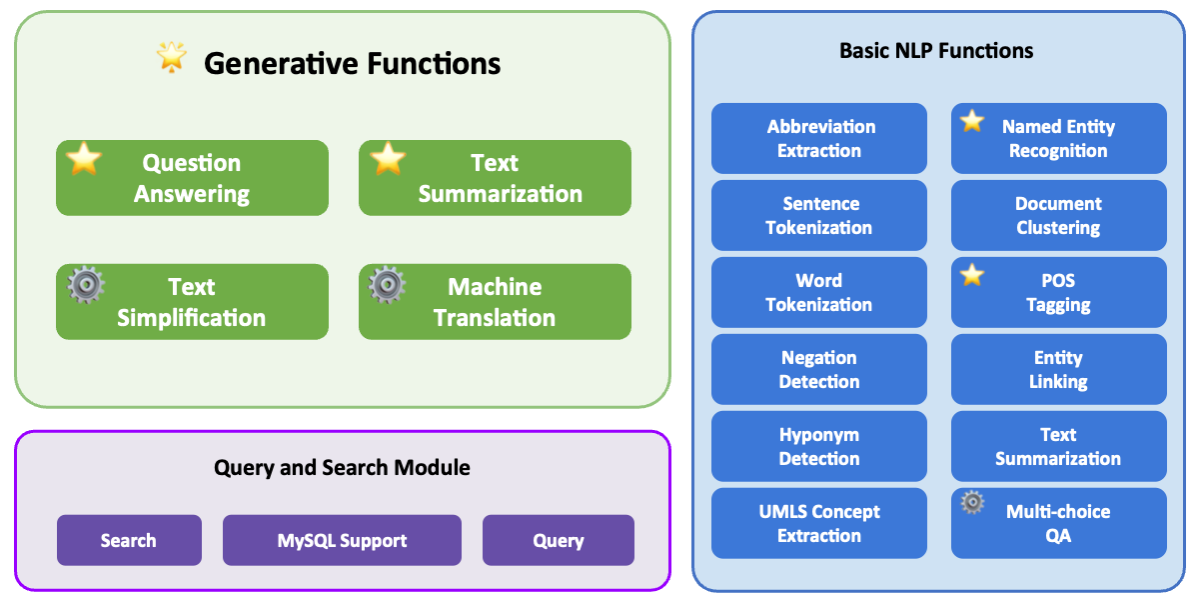
The overall architecture of Ascle. 
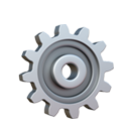
 indicates that we have our fine-tuned language models for this task. 
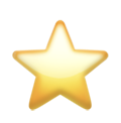
 indicates that we conducted evaluations for this task. POS: Parts-Of-Speech; QA: Question-Answering; UMLS: Unified Medical Language System.

Ascle consists of 3 modules, with the core module being the “generative functions,” including 4 challenging generation tasks: question-answering, text summarization, text simplification, and machine translation, covering a variety of application scenarios in health care. In addition, Ascle integrates 12 basic NLP functions, as well as query and search capabilities for clinical databases. The overall architecture of Ascle is shown in Figure 1. This section will focus on introducing the core module of Ascle—Generative Functions. For more information on basic NLP functions and query and search functions within Ascle, please refer to Multimedia Appendix 2 and Multimedia Appendix 3, respectively.

### Generative Functions

Ascle offers a range of generative functions through pretrained and fine-tuned language models, all of which are publicly available for user access. In the following sections, we will introduce these powerful generative functions separately.

#### Question-Answering

Question-answering is particularly crucial in health care [13]. When integrated into health care systems, it assumes roles, such as preconsultation and remote consultation, effectively coping with the exponential increase in patient load. Furthermore, specialized question-answering systems hold the potential to contribute to medical education [13,21].

In Ascle, we first provide an interface for medical LLMs, such as Baize-healthcare [23], allowing users to use them directly. In addition, we develop a RAG framework that uses UMLS with ranking techniques to enhance LLMs in generating long-form answers [21]. Specifically, when receiving a query, the RAG framework first extracts medical entities within the query automatically and then retrieves related triplets from UMLS for each extracted entity. A triplet consists of 2 medical concepts and the relation between them, that is, (Myopia, clinically_associated_with, HYPERGLYCEMIA). Subsequently, the RAG framework uses ranking and reranking techniques to refine the ordering of these triples. Finally, the query and the retrieved triplets would be prompted to LLM for inference. For more details about the RAG framework, please refer to Multimedia Appendix 4. We apply this framework to the GPT (OpenAI) [24] and LLaMA (Meta) [25] series of LLMs.

We conducted evaluations on 4 medical QA data sets, including LiveQA [26], ExpertQA (Med & Bio) [27], and MedicationQA [28]. LiveQA consists of health questions submitted by consumers to the National Library of Medicine. It includes a training set with 634 QA pairs and a test set comprising 104 QA pairs, and the test set was used for evaluation. ExpertQA is a high-quality long-form QA data set covering multiple fields, along with answers verified by domain experts. Among them, we used 504 medical questions (Med) and 96 biology (Bio) questions for evaluation. MedicationQA includes 690 drug-related consumer questions along with information retrieved from reliable websites and scientific papers.

Additionally, considering that automated metrics cannot effectively assess the quality of generated content, especially in terms of factuality, we performed a physician validation. A total of 50 question-answer pairs from LiveQA were randomly selected, with answers generated by Baize-healthcare. Subsequently, 2 health care professionals (1 resident and 1 attending specialist) rated these generated answers on the criteria of readability, relevancy, accuracy, and completeness, using a 5-point Likert scale.

#### Text Summarization

In health care, clinicians and researchers are confronted with an increasing amount of information, including literature, clinical notes, and more [29,30]. Text summarization is an important generation task, aiming to distill essential information from the overwhelming complexity of texts and compress it into a more concise format [31]. Through automatic text summarization, clinicians and researchers can efficiently acquire information, thereby avoiding information overload.

We provide an abstractive text summarization function and compare general pretrained summarization models, including Pegasus [32], BigBird [33], Bidirectional and Auto-Regressive Transformer (BART) [34], PRIMERA [35], as well as domain-specific models, such as SciFive [36] and BioBART [37], which make use of biomedical corpora. Furthermore, we chose PubMed [38], MIMIC Chest X-Ray (MIMIC-CXR [39]), and MEDIQA-AnS [40] data sets for evaluation. The PubMed data set consists of biomedical scientific publications from the PubMed database, where each input document is a scientific article with its abstract serving as the ground truth. We reported the evaluation results for the test set, which contains 1660 examples. MIMIC-CXR is a deidentified data set of chest radiographs with free-text radiology reports, and we used a subset of MIMIC-CXR that includes 2000 instances for evaluation. MEDIQA-AnS is a collection of 156 consumer health questions along with passages that contain relevant information. It supports both single-document and multiple-document summarization evaluation.

#### Text Simplification

Biomedical texts are typically laden with intricate terminologies, which can hinder the understanding of individuals without a clinical background [41]. In Ascle, the function of text simplification is to translate complex and technical biomedical texts into understandable content. This will enhance the comprehension and involvement of nonclinical individuals, including patients, enabling them to better engage with the information and participate in clinical decisions more effectively.

We finetuned and evaluated widely used pretrained language models on 3 data sets: eLife, PLOS [42], and MedLane [43]. This included 2 general models, BigBirdPegasus [32] and BART, as well as a biomedical-specific model, BioBART. The eLife and PLOS are shared task data released from the BioLaySumm 2023 Task 1, which contains biomedical journal articles with expert-written lay summaries. We evaluated the validation sets for eLife and PLOS, which contain 241 and 1376 examples, respectively. MedLane is a large-scale human-annotated data set containing professional-to-customer sentences selected from Medical Information Mart for Intensive Care III (MIMIC-III). For MedLane, we used the test set for evaluation, which includes 1016 instances.

#### Machine Translation

Language barriers pose difficulties for patients to access timely information and communicate effectively with health care providers, resulting in low-quality health care services [44]. Our machine translation function aims to translate the text from a source language into a target language in a clinical scenario. By fine-tuning pretrained language models on the medical corpus, Ascle supports machine translation from English (en) to 8 target languages: Spanish (es), French (fr), Romanian (ro), Czech (cs), German (de), Hungarian (hu), Polish (pl), and Swedish (sv). Here, we only emphasize the 8 languages fine-tuned on medical data, while other languages, such as English to Chinese, are supported by the pretrained language models.

We fine-tuned the existing MarianMT [45] and multilingual T5 [46] using UFAL Medical Corpus [47] which includes various medical text sources, such as titles of medical Wikipedia articles, medical term pairs, patents, and documents from the European Medicines Agency. During the preprocessing phase, we excluded general domain data from UFAL, such as parliamentary proceedings, and randomly shuffled the medical domain corpora, splitting them into 2 parts at a ratio of 85% and 15% for training and testing, respectively. We reported the results on the test set, the size of which varies from 111,779 to 407,388 depending on the different language pairs. Furthermore, for each language pair, we used all available parallel data to maximize the breadth and accuracy of our machine translation function.

### Ethical Considerations

This study used publicly available data sets and a restricted, deidentified data set. Access to the restricted data set was granted after the required training and certification, ensuring compliance with the data use agreement. No additional ethical review or informed consent was necessary, as human subjects or identifiable data were not directly involved. Two health care professionals voluntarily participated in the physician validation process without compensation. Data privacy and confidentiality were strictly maintained throughout the research, ensuring the protection of individual privacy while contributing to the advancement of the NLP toolkit for medical text generation.

## Results

### Overall Performance of Generation Tasks

In the question-answering task, we used ROUGE-L [48], BERTScore [49], MoverScore [50], and BLEURT [51] for a comprehensive evaluation, and used GPT-4 and LLaMA2-13b as the vanilla LLMs. As shown in Table 2, our RAG framework surpasses the zero-shot setting on all evaluation metrics for the LiveQA, ExpertQA-Bio, ExpertQA-Med, and MedicationQA data sets. Among them, the ROUGE-L score has increased by more than 18% on the ExpertQA-Bio data set.

For the text summarization task, we evaluated 5 pretrained language models on single-document summarization, as shown in Table 3. To ensure a fair comparison, we excluded the results of BioBART and SciFive on PubMed, as they were fine-tuned on this data set. It is worth noting that BART consistently demonstrated strong performance across 3 benchmarks, while BioBART only outperformed BART in 1 of the benchmarks. In addition, we evaluated the multidocument summarization task and discussed the differences between abstractive and extractive methods, as well as the limitations of evaluation metrics, which can be found in the Discussion section.

**Table 2:** For the evaluation of the question-answering task, we compared ROUGE-L, BERTScore, MoverScore, and BLEURT on the zero-shot and retrieval-augmented generation framework.

			LLaMa2-13b					GPT-4			
			ROUGE-L	BERTScore	MoverScore	BLEURT	ROUGE -L		BERTScore	MoverScore	BLEURT
**LiveQA^a^**											
	Z.S^b^		17.73	81.93	53.37	40.45	18.89		82.50	54.02	39.84
	RAG^c^		18.83^d^	82.79^d^	53.79^d^	40.59^d^	19.44^d^		83.01^d^	54.11^d^	40.55^d^
**ExpertQA^a^-Bio**											
	Z.S		23.26	84.38	55.58	44.65	23.00		84.50	56.15	44.53
	RAG		25.79^d^	85.18^d^	56.17^d^	45.20^d^	27.20^d^		85.83^d^	57.11^d^	45.91^d^
**ExpertQA^a^-Med**											
	Z.S		24.86	84.89	55.74	46.32	25.45		85.11	56.50	45.98
	RAG		27.49^d^	85.80^d^	56.58^d^	46.47^d^	28.08^d^		86.30^d^	57.32^d^	47.00^d^
**MedicationQA^a^**											
	Z.S		13.30	81.81	51.96	38.30	14.41		82.55	52.62	37.41
	RAG		14.71^d^	82.79^d^	52.59^d^	38.42^d^	16.19^d^		83.59^d^	53.30^d^	37.91^d^

^a^QA: question-answering.

^b^Z.S: zero-shot.

^c^RAG: retrieval-augmented generation framework.

^d^The superior score within the same data set.

**Table 3:** For the evaluation of the single-document summarization task, we compared ROUGE-1, ROUGE-2, ROUGE-L, and some results are derived from other papers [52].

	PubMed				MIMIC-CXR^a^				MEDIQA-AnS (p)				MEDIQA-AnS (s)		
	R-1	R-2	R-L	R-1		R-2	R-L	R-1		R-2	R-L	R-1		R-2	R-L
Pegasus	45.97	20.15	28.25	22.49		11.57	20.35	18.29		4.82	13.87	22.21		8.23	16.76
BigBird	46.32	20.65	42.33^b^	38.99		29.52	38.59	13.18		2.14	10.04	14.89		3.13	11.15
BART	48.35^b^	21.43^b^	36.90	41.70^b^		32.93^b^	41.16^b^	24.02^b^		7.20	17.09^b^	38.19		22.20	30.58
SciFive	—^c^	—	—	35.41		26.48	35.07	13.08		2.15	10.10	16.88		6.47	14.42
BioBART	—	—	—	41.61		32.90	41.00	22.58		7.49^b^	16.69	39.40^b^		24.64^b^	32.07^b^

^a^MIMIC-CXR: MIMIC Chest X-Ray database.

^b^The superior score within the same data set.

^c^Not applicable.

Regarding the text simplification task, we compared the performance of fine-tuned models and conducted an analysis of readability using the Flesch-Kincaid Grade Level (FKGL) score [53], as indicated in Table 4. For the eLife and PLOS data sets, the ground truth exhibits FKGL scores of 12 and 15, respectively. Interestingly, the BioBART model performs competitively in terms of ROUGE metrics, but fails to significantly reduce the difficulty of understanding, as evidenced by its FKGL score of 17 in both data sets. On the other hand, the BART model manages to slightly lower the FKGL score to 14 and 16 for eLife and PLOS, respectively. However, in the case of the MedLane data set, all methods appear to reach a similar level of complexity as the ground truth. This can be attributed to the data set’s shorter examples and potentially smaller vocabulary size, which limits the observed differences.

In the machine translation task, we fine-tuned the models across 8 languages, as illustrated in Table 5. After fine-tuning, the BLEU scores significantly improved, with the most substantial improvement observed in the “en-fr” language pair, increasing by over 61%. This enhancement can be attributed to the larger amount of training data available for “en-fr” (2,812,305 samples).

**Table 4:** For evaluation of the text simplification task, we compared ROUGE-1, ROUGE-2, ROUGE-L, and Flesch-Kincaid Grade Level score.

	eLife					PLOS					MedLane			
	R-1^a^	R-2^b^	R-L^c^	FKGL^d^	R-1		R-2	R-L	FKGL	R-1		R-2	R-L	FKGL
Ground Truth	—^e^	—	—	12	—		—	—	15	—		—	—	13
BigBirdPegasus	14.00	3.42	9.16	13^f^	18.92		4.79	12.54	17	74.96		65.37	74.56	13^f^
BART	16.16^f^	4.31^f^	10.19^f^	14	21.09		7.20	14.17	16^f^	83.25^f^		74.50^f^	82.99^f^	13^f^
BioBART	14.31	3.70	9.36	17	23.80^f^		7.83^f^	15.65^f^	17	82.89		74.26	82.65	13^f^

^a^R-1: ROUGE-1.

^b^R-2: ROUGE-2.

^c^R-L: ROUGE-L.

^d^FKGL: Flesch-Kincaid Grade Level.

^e^Not applicable.

^f^The superior score within the same data set.

**Table 5:** For the evaluation of the machine translation task, we evaluated the BLEU score on 8 language pairs.

	BLEU score							
	en-es^a^	en-fr^b^	en-ro^c^	en-cs^d^	en-de^e^	en-hu^f^	en-pl^g^	en-sv^h^
MarianMT	38.02	33.02	40.45	—^i^	—	—	—	—
F.T-MarianMT	41.64	43.72	43.88	—	—	—	—	—
F.T-mT5	45.88^j^	53.29^j^	47.28^j^	43.30	50.73	32.25	40.24	44.17

^a^en-es: English-Spanish.

^b^en-fr: English-French.

^c^en-ro: English-Romanian.

^d^en-cs: English-Czech.

^e^en-de: English-German.

^f^en-hu: English-Hungarian.

^g^en-pl: English-Polish.

^h^en-sv: English-Swedish.

^i^Not applicable.

^j^The superior score within the same data set.

### Physician Validation

**Figure 2:**
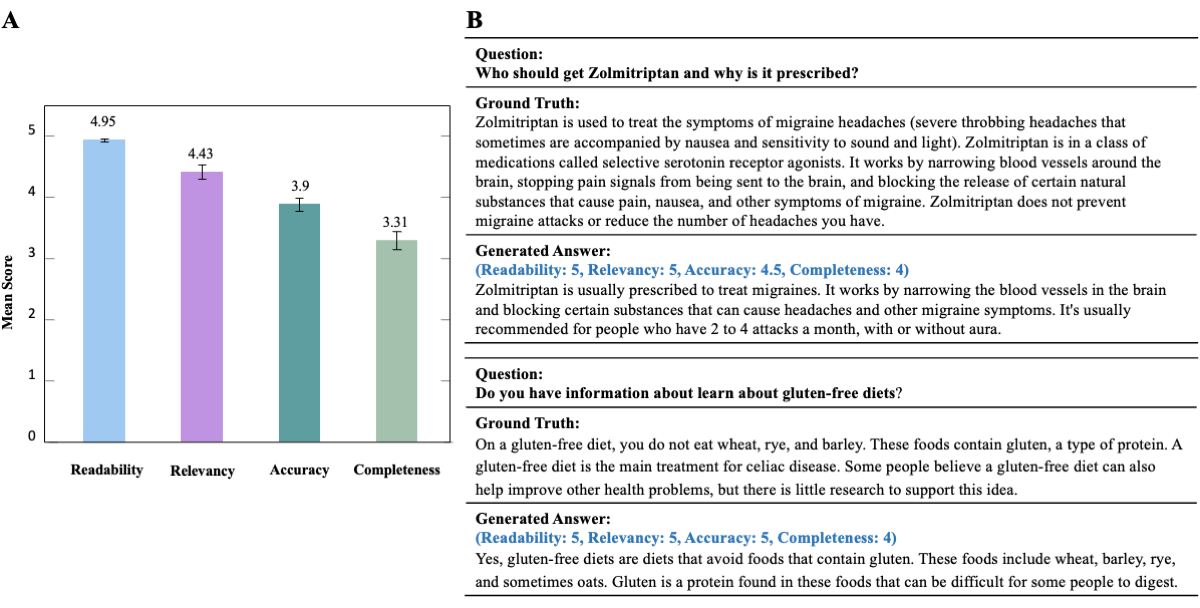
(A) Physician validation (readability, relevancy, accuracy, and completeness) for 50 question-answer pairs. (B) Two examples of generated answers with ground truth.

The results verified by the physicians are shown in Figure 2A. Detailed evaluation criteria can be found in Multimedia Appendix 5. The generated answers have good readability and relatively good relevancy, with scores of 4.95 and 4.43, respectively. In contrast, the completeness score is relatively lower (3.31). Figure 2B shows 2 cases. In the first case, compared with the ground truth, the generated answer does not point out that Zolmitriptan is used for treating acute migraines, nor does it indicate that it cannot be used to prevent migraine attacks or to reduce the frequency of headaches. In the second case, the generated answer does not mention that a gluten-free diet is the main treatment for celiac disease. We provide 2 additional cases in Multimedia Appendix 6.

In addition, we calculated the interevaluator agreement using percentage agreement for each criterion. A total of 2 health care professionals demonstrated a high level of consistency across all criteria, with the percentage agreement consistently exceeding 0.65.

## Discussion

### In-Depth Analysis of the Text Summarization Task

In the multidocument summarization task, we included models based on traditional methods, such as TextRank [54], as well as pretrained language models, such as BART, Pegasus, PRIMERA, and BioBART. We evaluated their performance using ROUGE scores on the MEDIQA-AnS data set, which consists of 156 examples, and the results are shown in Table 6. However, it is noteworthy that although TextRank outperforms almost all generative models in ROUGE scores, this does not necessarily indicate superior performance. As ROUGE scores are calculated based on the overlap between the generated content and reference summaries, and TextRank is an extractive summarization model, it tends to score higher by this measure.

**Table 6:** Evaluation for the multidocument summarization task.

	MEDIQA-AnS (p)				MEDIQA-AnS (s)		
	ROUGE-1	ROUGE-2	ROUGE-L	ROUGE-1		ROUGE-2	ROUGE-L
TextRank^a^	29.88	10.23	17.01	43.77		26.80	30.52
BART	24.56^b^	7.56^b^	17.18^b^	32.32^b^		15.42	24.03^b^
Pegasus	17.44	5.36	13.44	19.54		7.46	14.93
PRIMERA	16.66	4.89	12.68	21.78		9.77	16.85
BioBART	23.16	7.47	16.47	30.87		15.91^b^	23.66

^a^TextRank is only used as a reference for extractive summarization, so its scores are not compared with those of generative models.

^b^The superior score within the same data set.

While generative models possess semantic comprehension abilities, enabling them to distill complex information into an easy-to-understand format. As shown in Textbox 1, the summarizations generated by BART display well-structured patient information, with a brief description of events and corresponding conditions of the current patient (highlighted in blue), exhibiting high readability. In contrast, the summarizations produced by TextRank are less readable and include noise (highlighted in orange); the generated content is often a literal collage of text fragments. Despite TextRank achieving higher ROUGE scores, it lacks the ability to discern information and integrate it into coherent and readable content, showing significant limitations for practical use.

**Two MIMIC-III (parts) examples of the text summarization task, generated by BART and TextRank, respectively (we eliminated sensitive information).**

**BART**

- The patient is an XXX-year-old man with a history of a question of coronary artery disease, borderline diabetes mellitus. He was in his usual state of health until 11 p.m. last night when he experienced chest pain with radiation to his back, positive shortness of breath, positive diaphoresis, no vomiting, no lightheadedness. The patient had had a similar episode of chest pain and was taken to an XXX. He had successful angioplasty and stent of LAD and CX. He is a middle aged XXX man in no acute hypertensive distress. He has had anginal chest pain, which is similar to his presenting complaint, but without radiations to his Back. His blood pressure was 105/73, pulse 84, respiratory 21, O2 saturation 92% on 2 liters. His CPK was 594, The index was 7.7, and he was admitted to the hospital with a high blood pressure. His condition was described as “stable” and “normal” by the doctor. The doctor referred the patient to a cardiologist for further treatment. The cardiologist said the patient was in good condition and should be discharged in a few days.
- Patient has CABG complicated by postop bleed and pleural effusion with discharge to [**Hospital1 **] Rehabilitation presents with abdominal pain. Zosyn was given in the ED. Patient was otherwise doing well and was to go back to rehab to finish his course of Cipro and Flagyl on [**5-17**]. Patient was last seen normal sometime last evening. He woke up and noticed that the left side of his body felt “numb”. He was not aware of any other
  neurological weakness, and mostly complained of being very tired. He denied any new vision problems, did not have a headache. He sounded somewhat slurred but did not feel as if his speech was changed significantly. He felt sleepy but able to sustain attention, currently apparently in no distress. He was on standing. Plavix and [**State **] which had been held for the last few days (at least since the 14), since he had the percutaneous drainage. He did not. feel that the weakness had progress and reported that he felt the sense of numbness was starting to improve and had some difficulty squeezing an examiners hand. He is a retired postal worker. He lives with wife and son who is a chiropractor.

**TextRank**

- Admission Date: XXX Discharge Date: XXX Date of Birth: Sex: M Service: CCU-6HISTORY OF PRESENT ILLNESS: The patient is a XXX-year-old man with a history of a question of coronary artery disease, status post myocardial infarction in [**December 2175**], hypertension, borderline diabetes mellitus who was in his usual state of health until 11 p.m. last night when, while [**4-12**] midsternal pressure like chest pain with radiation to back, positive shortness of breath, positive diaphoresis, positive nausea, no vomiting, no lightheadedness. Mucous membranes moist. Oropharynx clear. NECK:  No jugular venous distention, no carotid bruits. CARDIOVASCULAR:  Regular rate, S1, S2, artificial S1 gallop and balloon pump, no murmurs or rubs. LUNGS: Bibasilar rales, left greater than right. ABDOMEN:  Normoactive bowel sounds, nontender, nondistended. EXTREMITIES:  No cyanosis, clubbing or edema. NEUROLOGIC:  Alert and oriented x3.LABS AT OUTSIDE HOSPITAL:  CPK was 304, troponin 1.75.Electrocardiogram at 1:23 a.m. was normal sinus rhythm at101, normal axis deviation, 2 to [**Street Address(2) 1755**] elevation V1 to V5,Q V3, AVF.LABS AT [**Hospital6 **] AT 8 A.M.:  CBC- white blood cells 11.2, hemoglobin 13.0, hematocrit 36.7,platelets 232. CARDIOVASCULAR: Coronary artery disease: Three vessel disease with successful intervention on LAD and left circumflex, but RCA not done secondary to good collateral. The patient was continued on aspirin 325 qd.
- Of note he was on standing Plavix and [**State **] which had been held for the last few days (atleast since the 14), since he had the percutaneous drainage. The patient was otherwise doing well and was to go back to rehab to finish his course of Cipro and Flagyl on [**5-17**].Past Medical History: coronary artery disease s/p right coronary artery stent x2([**10-3**], [**3-4**]), hypertension, hyperlipidemia, chronic obstructive pulmonary disease, asbestos exposure, chronic back pain, insomnia and obstructive sleep apnea (untreated)PSH:[**2144-4-21**]Endoscopic, minimally invasive, off pump coronary artery bypass graft x1 with left internal mammary artery to left anterior descending artery.[**2144-4-21**]Re-exploration for bleeding, post coronary artery bypass grafting. Social History: Lives with wife. Exposure to asbestos. Defers all medical decisions to son who is a chiropractor. Occupation: retired postal worker. Tobacco: 3 PPD x 30 years, quit 45 years ago ETOH: None Family History: Non-contributory to cholecystitis. Physical Exam: Physical Exam: Vitals: T: 97.9  P:75  R: 16  BP:128/73  SaO2:96 General: Awake, felt sleepy but able to sustain attention, poor historian currently.

### System Usage

**Figure 3:**
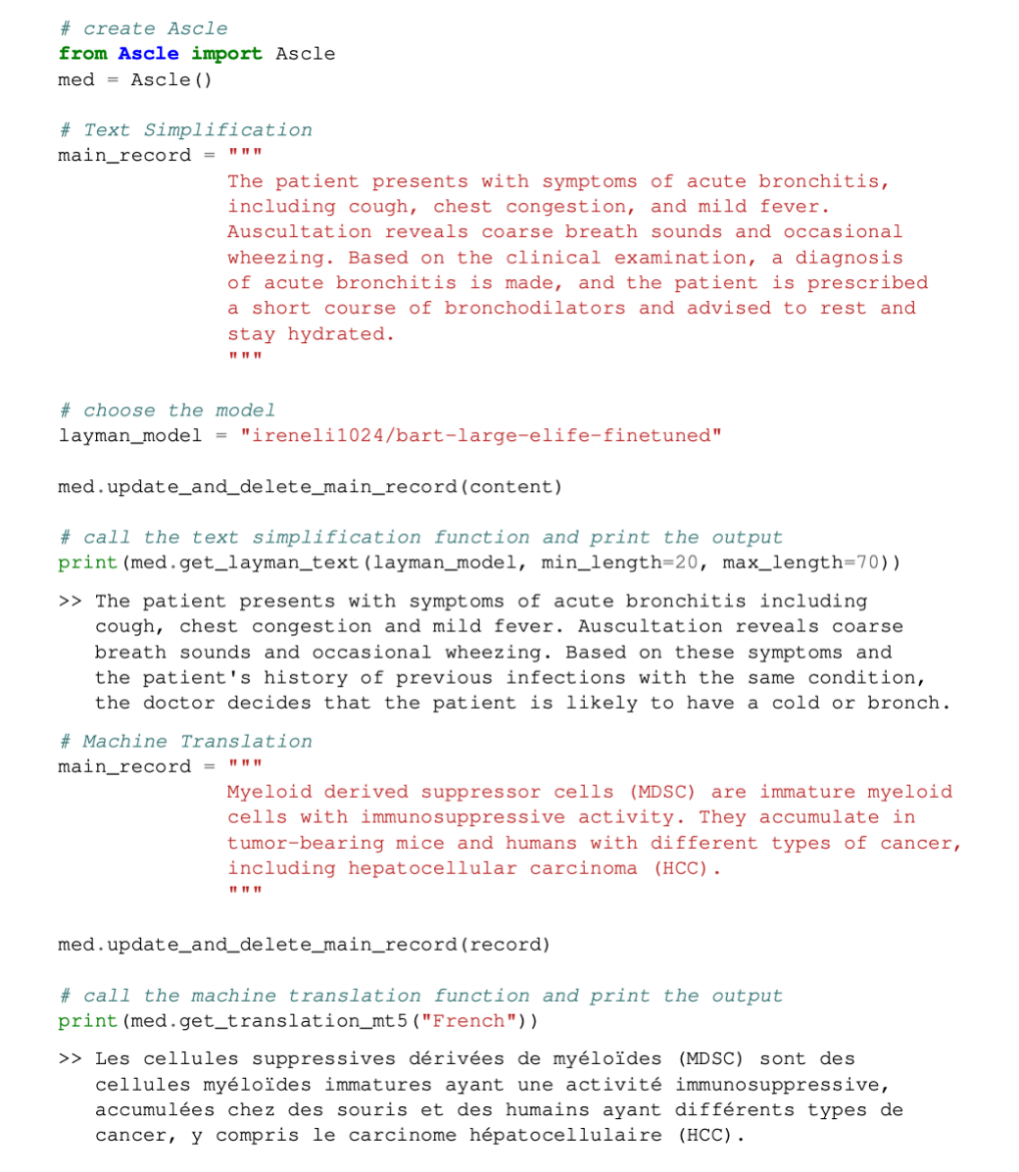
Demonstration of system usage. We show two use cases: Text Simplification and Machine Translation.

Ascle provides an easy-to-use approach for biomedical researchers and clinical staff. Users can efficiently use it by merely inputting text and calling the required functions. Figure 3 illustrates 2 use cases.

### Estimated Inference Time and Computational Resources

As shown in Table 7, we list the estimated inference time and computational resources required for the 4 generative tasks in Ascle. It is worth noting that the inference time is specific to our experimental settings, and the actual inference time for users may vary depending on the length of the input text and the computational resources used. For the question-answering task, GPT’s response time is faster compared with LLaMA2-13b. However, it is important to mention that LLaMA2-13b was not deployed with quantization, and with quantization, the required inference time and computational resource requirements would be reduced.

**Table 7:** Estimated inference time and computational resources required for the generative tasks in Ascle.

Tasks	Estimated inference time	Computational resource
Question Answering	LLaMA2-13b: <60 s/item^a^ GPT4: <15 s/item	LLaMA2-13b: 4 * NVIDIA A100 GPU GPT4: OpenAI API^b^
Text Summarization	<2 s/item	1 * NVIDIA V100 GPU
Text Simplification	<2 s/item	1 * NVIDIA V100 GPU
Machine translation	<2 s/item	1 * NVIDIA V100 GPU

^a^s/item: seconds per item.

^b^API: application programming interface.

### Clinicians’ Use of Ascle

To evaluate the ease of usability of Ascle for clinicians, we report the time required for 2 clinicians with different backgrounds to use the package after receiving guidance. The backgrounds of the clinicians are as follows: (1) physician 1: Singapore General Hospital, senior resident, 7 years of working experience, has a basic level of programming knowledge, and is able to perform basic statistical analyses; and (2) physician 2: SengKang General Hospital, senior consultant, 15 years of working experience, and has no programming knowledge.

Both clinicians received guidance on using Ascle, including setting up a virtual environment and accessing models from Hugging Face. The entire guidance process took about 10 minutes, after which both clinicians could independently and easily use Ascle and experiment with various generative functions without any issues. The main difficulty for the clinicians was setting up the virtual environment, as they lacked AI-specific knowledge. In response, Ascle provided a very simple virtual environment setup guideline. The clinicians’ experience further confirms the user-friendliness of Ascle.

### Limitations

In the case of generation tasks, we primarily chose automatic metrics for evaluation, such as ROUGE and BLEU scores. However, these metrics cannot effectively assess factual correctness [55] and may not align with human preference [56]. While human evaluation serves as an invaluable aspect in assessing the performance of the model, its incorporation may pose certain challenges due to various factors, including budget constraints.

### Future Work

Recent LLMs have shown great potential in generative applications especially its superior zero- and few-shot performance [13,57,58]. Despite this, the generated content can be unfaithful, inconsistent, and biased [21,55,59,60]. We plan to thoroughly evaluate LLMs and extend to Ascle in the future. Meanwhile, we will strengthen the ethical review of these generative AI techniques to ensure their application truly and responsibly benefits biomedical researchers and health care professionals [61,62].

### Conclusions

We introduce Ascle, a comprehensive NLP toolkit designed specifically for medical text generation. For the first time, it integrates 4 challenging generative functions, including question-answering, text summarization, text simplification, and machine translation. Our research fills the gap of existing toolkits for generative tasks, which holds significant implications for the entire medical domain. Ascle boasts remarkable flexibility, allowing users to access a variety of cutting-edge pretrained language models. Meanwhile, it stands as a user-friendly toolkit, ensuring ease of use even for clinical staff without a technical background. We will continue to maintain and extend Ascle.

## Appendix

Multimedia Appendix 1 32 Fine-tuned language models and 27 benchmarks in Ascle.

Multimedia Appendix 2 Basic natural language processing functions in Ascle.

Multimedia Appendix 3 Query and search module in Ascle.

Multimedia Appendix 4 The Retrieval-Augmented Generation framework in Ascle – KG-Rank.

Multimedia Appendix 5 Evaluation criteria for physician validation.

Multimedia Appendix 6 Case study.

## Abbreviations

BART: Bidirectional and Auto-Regressive Transformer
BERT: Bidirectional Encoder Representations from Transformers
FKGL: Flesch-Kincaid Grade Level
LLM: large language model
NLP: natural language processing
RAG: Retrieval-Augmented Generation
UMLS: Unified Medical Language System
