# Supplementary material for: Ascle—A Python Natural Language Processing Toolkit for Medical Text Generation: Development and Evaluation Study
Source: J Med Internet Res. 2024 Oct 3;26:e60601. doi: 10.2196/60601 (PMC11487205; doi:10.2196/60601)
Supplement: Multimedia Appendix 1 [file jmir_v26i1e60601_app1.docx]

**Multimedia Appendix 1**

**32 Fine-Tuned Language Models and 27 Benchmarks in Ascle**

In Table 1, we list the 32 fine-tuned language models of Ascle. We upload all of them to Hugging Face. All tasks were performed using one NVIDIA V100 GPU. For the multiple-choice QA task, the training time for each model was between 1-3 hours. For the text simplification task, the training time for each model was between 8-12 hours. For the machine translation task, the training time for each model was between 8-12 hours. Please note that the specific time is related to factors such as dataset size, computational resources, hyperparameter settings, etc.

Table 1. 32 fine-tuned language models of Ascle.

| **Tasks** | **Base Model** | **Fine-Tuned Data** | **Hugging Face Access** |
| --- | --- | --- | --- |
| Multi-choice QA | BioBERT | HEADQA | <https://huggingface.co/li-lab/ascle-BioBERT-finetune-HEADQA> |
|  | ClinicalBERT | HEADQA | <https://huggingface.co/li-lab/ascle-ClinicalBERT-finetune-HEADQA> |
|  | SapBERT | HEADQA | <https://huggingface.co/li-lab/ascle-SapBERT-finetune-HEADQA> |
|  | PubMedBERT | HEADQA | <https://huggingface.co/li-lab/ascle-PubMedBERT-finetune-HEADQA> |
|  | GatorTron | HEADQA | <https://huggingface.co/li-lab/ascle-GatorTron-finetune-HEADQA> |
|  | BioBERT | MedMCQA-w-context | <https://huggingface.co/li-lab/ascle-BioBERT-finetune-MedMCQA-w-context> |
|  | ClinicalBERT | MedMCQA-w-context | <https://huggingface.co/li-lab/ascle-ClinicalBERT-finetune-MedMCQA-w-context> |
|  | SapBERT | MedMCQA-w-context | <https://huggingface.co/li-lab/ascle-SapBERT-finetune-MedMCQA-w-context> |
|  | PubMedBERT | MedMCQA-w-context | <https://huggingface.co/li-lab/ascle-PubMedBERT-finetune-MedMCQA-w-context> |
|  | GatorTron | MedMCQA-w-context | <https://huggingface.co/li-lab/ascle-GatorTron-finetune-MedMCQA-w-context> |
|  | BioBERT | MedMCQA-wo-context | <https://huggingface.co/li-lab/ascle-BioBERT-finetune-MedMCQA-wo-context> |
|  | ClinicalBERT | MedMCQA-wo-context | <https://huggingface.co/li-lab/ascle-ClinicalBERT-finetune-MedMCQA-wo-context> |
|  | SapBERT | MedMCQA-wo-context | <https://huggingface.co/li-lab/ascle-SapBERT-finetune-MedMCQA-wo-context> |
|  | PubMedBERT | MedMCQA-wo-context | <https://huggingface.co/li-lab/ascle-PubMedBERT-finetune-MedMCQA-wo-context> |
|  | GatorTron | MedMCQA-wo-context | <https://huggingface.co/li-lab/ascle-GatorTron-finetune-MedMCQA-wo-context> |
| Text Simplification | BART | eLife | <https://huggingface.co/li-lab/ascle-bart-large-elife-finetuned> |
|  | BioBART | eLife | <https://huggingface.co/li-lab/ascle-biobart-v2-base-elife-finetuned> |
|  | BigBirdPegasus | eLife | <https://huggingface.co/li-lab/ascle-bigbird-pegasus-large-pubmed-elife-finetuned> |
|  | BART | PLOS | <https://huggingface.co/li-lab/ascle-bart-large-PLOS-finetuned> |
|  | BioBART | PLOS | <https://huggingface.co/li-lab/ascle-biobart-v2-base-plos-finetuned> |
|  | BigBirdPegasus | PLOS | <https://huggingface.co/li-lab/ascle-bigbird-pegasus-large-pubmed-plos-finetuned> |
| Machine Translation | mT5 | UFAL (en_es) | <https://huggingface.co/li-lab/ascle-en-es-UFAL-medical> |
|  | mT5 | UFAL (en_fr) | <https://huggingface.co/li-lab/ascle-en-fr-UFAL-medical> |
|  | mT5 | UFAL (en_ro) | <https://huggingface.co/li-lab/ascle-en-ro-UFAL-medical> |
|  | mT5 | UFAL (en_cs) | <https://huggingface.co/li-lab/ascle-en-cs-UFAL-medical> |
|  | mT5 | UFAL (en_de) | <https://huggingface.co/li-lab/ascle-en-de-UFAL-medical> |
|  | mT5 | UFAL (en_hu) | <https://huggingface.co/li-lab/ascle-en-hu-UFAL-medical> |
|  | mT5 | UFAL (en_pl) | <https://huggingface.co/li-lab/ascle-en-pl-UFAL-medical> |
|  | mT5 | UFAL (en_sv) | <https://huggingface.co/li-lab/ascle-en-sv-UFAL-medical> |
|  | MarianMT | UFAL (en_es) | <https://huggingface.co/li-lab/ascle-en-es-UFAL-MarianMT> |
|  | MarianMT | UFAL (en_fr) | <https://huggingface.co/li-lab/ascle-en-fr-UFAL-MarianMT> |
|  | MarianMT | UFAL (en_ro) | <https://huggingface.co/li-lab/ascle-en-ro-UFAL-MarianMT> |

In Table 2, we list the 27 benchmarks evaluated in Ascle.

Table 2. 27 evaluated benchmarks in Ascle.

| **Tasks** | **Benchmarks** |
| --- | --- |
| Question Answering | LiveQA |
|  | ExpertQA-Bio |
|  | ExpertQA-Med |
|  | MedicationQA |
| Text Summarization | PubMed |
|  | MIMIC-CXR |
|  | MEDIQA-AnS (p) |
|  | MEDIQA-AnS (s) |
| Text Simplification | eLife |
|  | PLOS |
|  | MedLane |
| Machine Translation | UFAL en-es |
|  | UFAL en-fr |
|  | UFAL en-ro |
|  | UFAL en-cd |
|  | UFAL en-de |
|  | UFAL en-hu |
|  | UFAL en-pl |
|  | UFAL en-sv |
| Multi-choice QA | HEADQA |
|  | MedMCQA-w-context |
|  | MedMCQA-wo-context |
| POS Tagging | CRAFT-SA |
|  | GENIA |
| Named Entity Recognition | NCBI-disease |
|  | BC5CDR-disease |
|  | BC5CDR-chem |
