# Supplementary material for: Ascle—A Python Natural Language Processing Toolkit for Medical Text Generation: Development and Evaluation Study
Source: J Med Internet Res. 2024 Oct 3;26:e60601. doi: 10.2196/60601 (PMC11487205; doi:10.2196/60601)
Supplement: Multimedia Appendix 2 [file jmir_v26i1e60601_app2.docx]

**Multimedia Appendix 2**

**Basic NLP Functions in Ascle**

Ascle integrates many third-party libraries and supports up to 12 basic functions, including abbreviation extraction, sentence tokenization, word tokenization, negation detection, hyponym detection, UMLS concept extraction, named entity recognition, document clustering, POS tagging, entity linking, text summarization (extractive methods), and multi-choice QA, as shown in Table 1.

Table 1. 12 basic NLP functions in Ascle.

| **Functions** | **Libraries** |
| --- | --- |
| Abbreviation Extraction | scispaCy |
| Sentence Tokenization | MedspaCy, scispaCy, Stanza Biomed |
| Word Tokenization | MedspaCy, scispaCy, Stanza Biomed |
| Negation Detection | scispaCy |
| Hyponym Detection | scispaCy |
| UMLS Concept Extraction | scispaCy |
| Named Entity Recognition | MedspaCy, scispaCy, Stanza Biomed |
| Document Clustering | GloVe, Transformers |
| POS Tagging | MedspaCy, scispaCy, Stanza Biomed |
| Entity Linking | MedspaCy |
| Text Summarization | summa |
| Multi-choice QA | Transformers |

***Multi-choice QA***

For the multi-choice QA task, we conducted comparative analysis on five biomedical and clinical pre-trained language models, including BioBERT, ClinicalBERT, SapBERT, GatorTron, and PubMedBERT. These models were fine-tuned and evaluated on the Head-QA and MedMCQA datasets. HEAD-QA covers questions in six topics: medicine, nursing, psychology, chemistry, pharmacology, and biology, all of which are derived from professional position exams within the Spanish healthcare system. We conducted evaluation on the test set, which contains 2,724 instances.

MedMCQA is a larger dataset that covers 2,400 healthcare topics and 21 medical subjects. Due to the lack of labels in the MedMCQA test set, we utilized 4,183 questions from the validation set for evaluation. We employed accuracy score as the evaluation metric, as shown in Supplementary Figure 1. Among these, PubMedBERT excels on HEAD-QA and MedMCQA (without context) with accuracy rates of 42.52% and 46.59% respectively. However, SapBERT, PubMedBERT and GatorTron-base achieve a very similar performance on MedMCQA (with context), especially GatorTron-base emerges as the superior performer, boasting an accuracy of 64.93%.


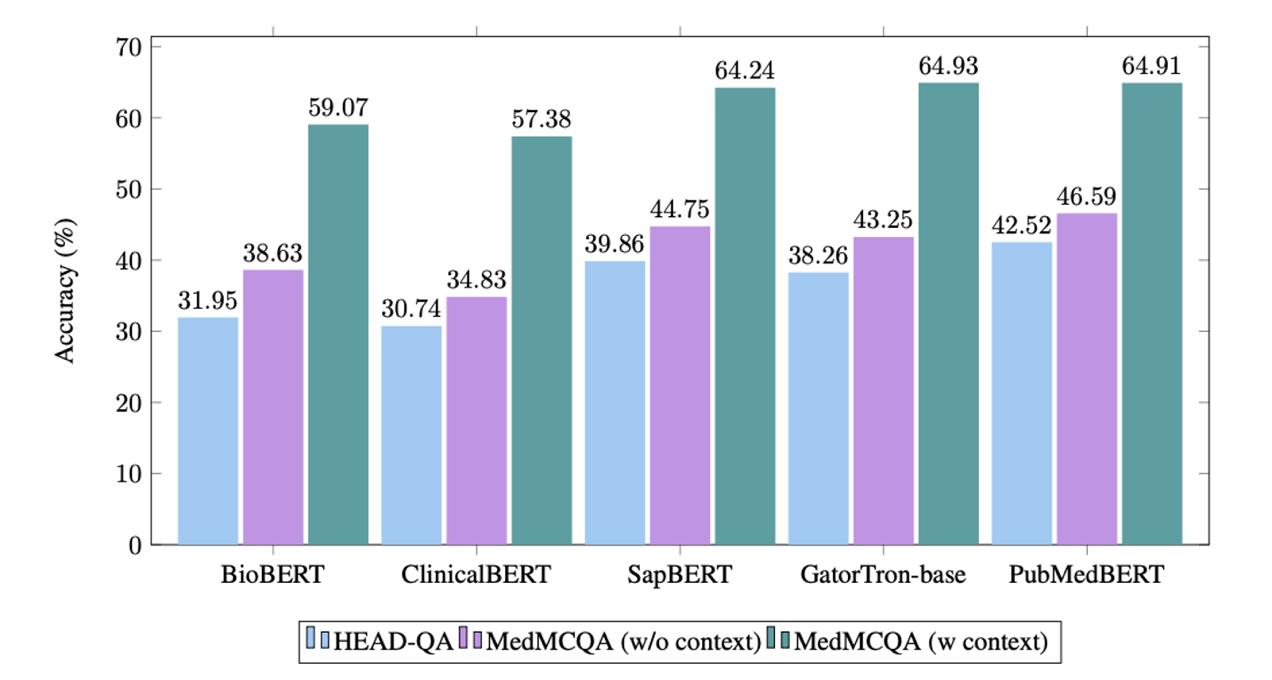
Figure 1. Evaluation for the multiple-choice QA task.

***POS Tagging***

For the POS tagging task, we conducted evaluations on the 2019 CRAFT Shared Task (CRAFT-SA) and GENIA corpus. The testing case numbers are 9,069 and 2,036 respectively. We chose to test on various scispaCy models, a pretrained open-source model (flair/pos-english), and four Stanza Biomed models, with the results shown in Supplementary Figure 2. We concluded that scispaCy models are better on GENIA significantly, but on CRAFT, they all have a comparable performance.
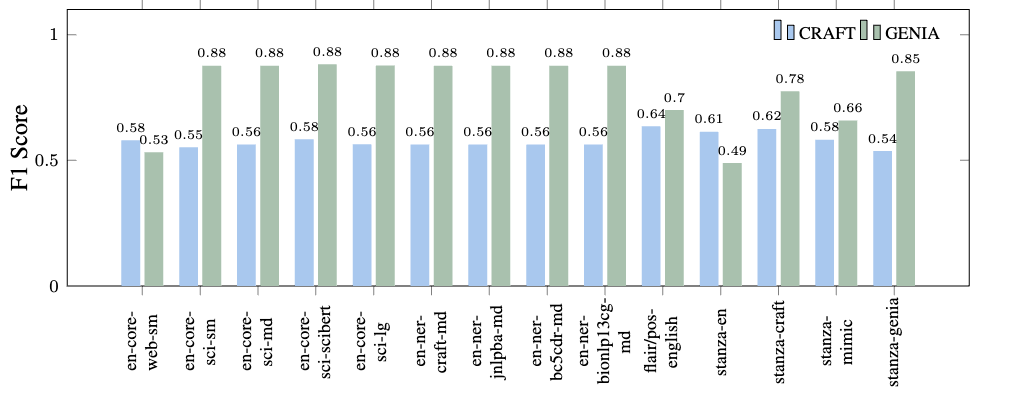


Figure 2. Evaluation for the POS tagging task.

***Named Entity Recognition***

We chose NCBI disease corpus and BC5CDR (which contains both disease and chemical entity types) datasets for evaluation. The test sets for these two datasets consist of 941 and 4,797 instances, respectively. We compared seven different models from scispaCy and Stanza Biomed, as shown in Table 2. Stanza models exhibit superior performance, but it is noteworthy that these models were specifically pre-trained for these datasets, whereas in the scispaCy, only "scispaCy-bc5cdr-md" was specifically pre-trained on the BC5CDR dataset.

Table 2. Evaluation for the NER task.

|  | NCBI-disease | | | BC5CDR-disease | | | BC5CDR-chem | | |
| --- | --- | --- | --- | --- | --- | --- | --- | --- | --- |
|  | R | P | F1 | R | P | F1 | R | P | F1 |
| scispaCy-sci-sm | 61.88 | 8.07 | 14.28 | 74.55 | 8.76 | 15.68 | 73.24 | 10.48 | 18.33 |
| scispaCy-sci-md | 62.50 | 7.98 | 14.16 | 76.60 | 8.89 | 15.94 | 77.51 | 10.96 | 19.20 |
| scispaCy-bc5cdr-md | 51.56 | 52.38 | 51.97 | 76.24 | 36.39 | 49.27 | 84.70 | 49.21 | 62.25 |
| Stanza-default | **83.44** | 82.83 | **83.13** | 76.51 | 78.10 | 77.30 | 80.58 | 86.01 | 83.20 |
| Stanza-mimic | 76.98 | **84.36** | 80.50 | 75.95 | 80.02 | 77.93 | 76.06 | 86.74 | 81.05 |
| Stanza-craft | 79.69 | 84.16 | 81.86 | **81.04** | 79.95 | 80.49 | **85.89** | 88.33 | **87.09** |
| Stanza-genia | 76.56 | 83.52 | 79.89 | 80.42 | **81.29** | **80.85** | 82.95 | **88.49** | 85.63 |
