# Supplementary material for: Ascle—A Python Natural Language Processing Toolkit for Medical Text Generation: Development and Evaluation Study
Source: J Med Internet Res. 2024 Oct 3;26:e60601. doi: 10.2196/60601 (PMC11487205; doi:10.2196/60601)
Supplement: Multimedia Appendix 3 [file jmir_v26i1e60601_app3.docx]

**Multimedia Appendix 3**

**Query and Search Module in Ascle**

Ascle provides user-friendly query and search functions on text corpora:

(1) *MySQL Support for MIMIC database*: The data tables (i.e., NOTEEVENTS.TSV) were indexed into a MySQL database, and user-friendly interfaces were provided for basic statistical functions, such as obtaining the count of patients, documents, and sentences.

(2) *Query*: We implemented a range of straightforward query functions. For instance, users could retrieve a specified number of patient records or notes by using their respective IDs.

(3) *Search*: The effectiveness of search functionality within unstructured text was of paramount importance. To address this, we integrated keyword search capabilities supported by multiple libraries, thus enabling swift and targeted searches.
