# Supplementary material for: Ascle—A Python Natural Language Processing Toolkit for Medical Text Generation: Development and Evaluation Study
Source: J Med Internet Res. 2024 Oct 3;26:e60601. doi: 10.2196/60601 (PMC11487205; doi:10.2196/60601)
Supplement: Multimedia Appendix 4 [file jmir_v26i1e60601_app4.docx]

**Multimedia Appendix 4**

**The RAG Framework in Ascle – KG-Rank**

As shown in Figure 1, this is the RAG framework (KG-Rank) used in Ascle to enhance long-form medical question answering.

Figure 1. An illustration of KG-Rank Framework

We define the external knowledge graph (KG) as *G* = *(V, E)*, where *V* represents the set of entities and *E* represents the set of structural relations. For the medical QA task, we choose UMLS as the primary medical KG. In the first step, we extract key entities and find mappings from the external KG. Specifically, for the given query *Q*, we apply a medical NER prompt $P_{MedNER}$ to identify related medical entities *E_Q*, and then we map each entity *e_i* ∈ *E_Q* to the corresponding entity in the knowledge graph *G.* After identifying the corresponding entities *E_Q'*, we retrieve their one-hop relations from the KG:

*E_Q'* = {*e_i'* ∈ *V* | ∃*e_i* ∈ *E_Q*, *e_i* ↦ *e_i'*}

Within UMLS, there exists extensive relational information, where one entity may be associated with thousands of one-hop relations. Consequently, we propose ranking and re-ranking techniques to extract the most relevant information. After that, the query and retrieved information will be prompted to LLMs for inference.

For more details and the prompts used in the RAG framework, please refer to: [KG-Rank: Enhancing Large Language Models for Medical QA with Knowledge Graphs and Ranking Techniques](https://arxiv.org/abs/2403.05881)
