# Supplementary material for: Ascle—A Python Natural Language Processing Toolkit for Medical Text Generation: Development and Evaluation Study
Source: J Med Internet Res. 2024 Oct 3;26:e60601. doi: 10.2196/60601 (PMC11487205; doi:10.2196/60601)
Supplement: Multimedia Appendix 5 [file jmir_v26i1e60601_app5.docx]

**Multimedia Appendix 5**

**Evaluation Criteria for Physician Validation**

Table 1. Evaluation criteria for physician validation.

| **Readability**  The quality of the answer text, ignoring the input.  1 (bad): The text is highly difficult to read, full of grammatical errors, and lacks coherence and clarity.  2: The text is somewhat difficult to read, and there are occasional grammatical errors. The coherence and clarity could be improved.  3: The text is moderately easy to read, but there are noticeable grammatical errors, and some parts lack coherence and clarity.  4: The text is fairly easy to read, with only a few minor grammatical errors. Overall coherence and clarity are good, but there is room for improvement.  5 (good): The text is easy to read, well-structured, and flows naturally. |
| --- |
| **Relevancy**  The pertinence of the answer to the posed question.  1 (bad): The answer is entirely off-topic and does not address the question at all.  2: The answer somewhat addresses the question but contains a significant amount of irrelevant information.  3: The answer is moderately relevant to the question but could be more focused.  4: The answer is mostly relevant with only minor deviations from the topic.  5 (good): The answer directly addresses the question and stays on topic throughout. |
| **Accuracy**  The correctness and truthfulness of the information provided in the answer.  1 (bad): The answer contains entirely incorrect or misleading information.  2: The answer contains several inaccuracies or misleading statements.  3: The answer is somewhat accurate but has noticeable errors.  4: The answer is mostly accurate with only minor errors.  5 (good): The answer is entirely accurate and trustworthy. |
| **Completeness**  The extent to which the answer covers all aspects of the question **(compared with the ground truth)**.  1 (bad): The answer barely touches on the topic and leaves out most of the necessary information.  2: The answer covers some aspects of the question but misses several key points.  3: The answer provides a moderate amount of information but could be more comprehensive.  4: The answer is fairly comprehensive but misses a few minor details.  5 (good): The answer thoroughly addresses all aspects of the question and leaves no stone unturned. |
