# Supplementary material for: Ascle—A Python Natural Language Processing Toolkit for Medical Text Generation: Development and Evaluation Study
Source: J Med Internet Res. 2024 Oct 3;26:e60601. doi: 10.2196/60601 (PMC11487205; doi:10.2196/60601)
Supplement: Multimedia Appendix 6 [file jmir_v26i1e60601_app6.docx]

**Multimedia Appendix 6**

**Case Study**

Based on the physicians' ratings, we selected two typical cases: In the first case, the generated answer is entirely consistent with the ground truth, achieving a score of 5 in readability, relevancy, accuracy, and completeness. In the second case, although the generated answer scored 5 in readability and scored 4 in relevancy, it is incorrect and completely contrary to the ground truth, receiving the lowest scores in accuracy and completeness.

Table 6. Two cases for human validation.

| **Question:**  What should I do if I forget a dose of Amphetamine? |
| --- |
| **Ground Truth:**  Take the missed dose as soon as you remember it. However, if it is almost time for the next dose, skip the missed dose and continue your regular dosing schedule. Do not take a double dose to make up for a missed one.) |
| **Generated Answer by Baize-healthcare**  **(Readability: 5, Relevancy: 5, Accuracy: 5, Completeness: 5)**  If you forget a dose of Amphetamine, you should take it as soon as you remember. However, if it is almost time for your next dose, skip the missed dose and continue with your regular dosing schedule. Do not take a double dose to make up for the missed one. |

| **Question:**  Is ring chromosome 20 syndrome inherited? (Also called: r(20) syndrome; ring 20 syndrome; ring chromosome 20; ring chromosome 20 epilepsy syndrome) |
| --- |
| **Ground Truth:**  **Ring chromosome 20 syndrome is almost never inherited.** A ring chromosome typically occurs as a random event during the formation of reproductive cells (eggs or sperm) or in early embryonic development. Often, the ring chromosome is present in only some of a person's cells. This situation is known as mosaicism. Most affected individuals have no history of the disorder in their families. However, at least one family has been reported in which a ring chromosome 20 was passed from a mother to her children.) |
| **Generated Answer by Baize-healthcare**  **(Readability: 5, Relevancy: 4, Accuracy: 1, Completeness: 1)**  **Yes, ring chromosome 20 syndrome is inherited in an autosomal recessive manner,** which means that a person must inherit two copies of the mutated gene (one from each parent) to develop the condition. |
